# Experiences of children and their caregivers affected by multidrug-resistant tuberculosis in Cape Town, South Africa

**DOI:** 10.1371/journal.pone.0323492

**Published:** 2025-05-19

**Authors:** Abenathi Mcinziba, Dillon T. Wademan, Klassina Zimri, Stephanie Jacobs, Khanyisa Mcimeli, H. Simon Schaaf, Anneke C. Hesseling, James A. Seddon, Thomas Wilkinson, Graeme Hoddinott

**Affiliations:** 1 Desmond Tutu TB Centre, Department of Paediatrics and Child Health, Faculty of Medicine and Health Sciences, Stellenbosch University, Cape Town, South Africa; 2 Department of Infectious Disease, Imperial College London, London, United Kingdom; 3 Health Economics Unit, School of Public Health and Family Medicine, University of Cape Town, Cape Town, South Africa; 4 School of Public Health, Faculty of Medicine and Health, The University of Sydney, Sydney, Australia; Management Sciences for Health (MSH), ETHIOPIA

## Abstract

**Background:**

Approximately 30,000 children (<15 years) develop multidrug-resistant (MDR) tuberculosis (TB) each year. MDR-TB severely impacts the lives of children and their families, yet data exploring their experiences are limited. We describe the experiences of children routinely treated for MDR-TB and their caregivers throughout their MDR-TB journeys in Cape Town, South Africa.

**Methods:**

We conducted a series of three in-depth qualitative interviews (48 interviews in total) with 17 children (<15 years) and/or their caregivers between April 2021 and September 2021. We selected children who had been routinely treated for MDR-TB between 2018 and 2021. We applied a deductive, thematic analysis to case summaries with illustrative examples from interviews.

**Findings:**

Children had negative experiences throughout their MDR-TB journey, before their diagnosis, during the diagnostic process, through treatment, and beyond treatment completion. Children and their caregivers experienced delays in acquiring accurate and timely MDR-TB diagnosis; stating lack of symptom recognition and repeated referrals between health facilities. Once on treatment, caregivers experienced challenges administering MDR-TB medication as children resisted taking their medications due to poor palatability, tolerability, and negative side effects. Some caregivers reported that, beyond treatment, children experienced extended physical challenges such as shortness of breath. Additionally, MDR-TB diagnosis and treatment negatively affected family life, as caregivers adjusted household spending toward foods that facilitated ingestion and mitigated side effects. Caregivers also juggled between attending to their children’s MDR-TB care and other household priorities.

**Conclusion:**

There are multifactorial challenges experienced by children and their caregivers throughout their MDR-TB journey. Research is needed to develop holistic interventions for child-caregiver-centred psychosocial support to mitigate the negative impact of MDR-TB on children and their caregivers through prevention, earlier diagnosis, and simpler, child-friendly regimens.

[1,1,1,2,3]

## Background

Tuberculosis (TB) is the leading infectious disease killer in the world, claiming 1.25 million lives in 2023 [1]. In 2023, an estimated 10.8 million people developed TB worldwide, including ~1.3 million children <15 years old [1]. South Africa is among 30 countries with the highest incidence of TB (468 per 100,000 population in 2022) and accounted for 16,534 children (<15 years of age) TB notifications [1]. South Africa’s TB epidemic is driven by several factors including low socio-economic status and high HIV coinfection burden [2,3].

Drug-susceptible TB can be effectively treated and cured, with treatment outcomes in children better than among adults [4–6]. However, if TB is undiagnosed and untreated, mortality and morbidity are substantial [7]. Drug-resistant (DR)-TB diagnosis and treatment are more challenging, with longer, less effective, less acceptable treatment, especially for children [8–10]. Each year, approximately 30,000 children develop multidrug-resistant (MDR) TB (i.e., TB disease caused by *Mycobacterium tuberculosis* resistant to at least isoniazid and rifampicin) [11,12].

Typically, treatment for MDR-TB in children is between nine to 24 months but recent guidelines have recommended six months treatment for non-severe cases [13]. Treatment can place a substantial financial burden on both affected families and the health system [14]. There have been notable recent improvements in MDR-TB treatment, including the availability of shorter, all-oral regimens that facilitate ambulatory care [15,16]. However, MDR-TB treatment for children lags in these innovations [17–19], and involves a complex regimen of drugs – many developed 60–70 years ago – with poor acceptability and substantial side effects [9,20,21]. MDR-TB among children may involve extended periods of hospitalization or other disruptions to family support [22]. Children’s MDR-TB treatment journeys are often highly distressing and many, including adolescents, experience psychosocial challenges such as social isolation, stigma, depression, and poor self-esteem [8,23,24].

However, the literature on children’s experiences of MDR-TB is limited, with a handful of qualitative studies with small sample sizes and where describing children’s experiences is often only a secondary aim or emergent finding [8,24,25]. This means that there are limited data to inform the operationalisation of more “child-centred” MDR-TB care, despite this being a priority. We aimed to close this gap with in-depth qualitative data, purpose-built to describe the experiences of children affected by MDR-TB in the high TB-burden setting of Cape Town, South Africa. Specifically, our objectives were to (a) describe children’s pathways to entering care, receiving diagnosis and treatment, (b) the impact of MDR-TB on their families and social lives, (c) the lingering post-treatment effects, and (d) to potentially inform future MDR-TB care evaluations among children.

## Methods

### Study design

We carried out a deductive thematic analysis of qualitative data collected within the MDR-TBKids project, which assessed the impact of paediatric MDR-TB on children and their caregivers prior and during the COVID-19 pandemic period. The MDR-TBKids project comprised an in-depth household survey of children (and their caregivers) aged <15 years of age treated for MDR-TB disease between January 2018 and November 2021. The nested qualitative component reported in this paper described these children’s experiences of MDR-TB care from their caregivers’ perspectives. The survey excluded older adolescents as those above 15 years of age are treated in adult care services in South Africa [13].

### Setting

The MDR-TBKids study took place in Cape Town, South Africa. Cape Town is the second largest metro in South Africa with a population of ∼4.7 million people [26]. A prospective surveillance study of children with confirmed TB attending a tertiary hospital in Cape Town, between 2013–2017, reported an estimated MDR-TB prevalence of between 7.1% and 8.9% [27].

Since 2018 many children have been treated as outpatients because injectable agents are rarely used. Many children (<15 years) with MDR-TB in Cape Town are referred to Brooklyn Chest Hospital (BCH) – a large referral hospital for TB patients in the Western Cape, South Africa. Children are referred for ongoing MDR-TB treatment following treatment initiation at Tygerberg Hospital (TBH) or other health facilities such as Red Cross Children’s War Memorial Hospital, Cape Town [27]. Most children attending BCH were discharged home during or after the completion of an initial phase of treatment. After that time, they were either managed exclusively in their local primary care clinic or returned for specialist follow-up appointments at TBH.

### Sampling and recruitment

Participants for the MDR-TBKids survey were sampled from those who had received care at Tygerberg Hospital or Brooklyn Chest Hospital and who expressed interest in taking part in the study. We recruited a purposive sub-sample of the MDR-TBKids participants, sampling for diversity in age, sex, and home language, between 30 November 2020 and 30 September 2021. This sampling technique enabled us to recruit and gain in-depth understanding and a diverse range of children’s experiences until we reached data saturation in this population group [28]. We asked and accessed participants’ diagnostic and treatment records during this period. Potential participants were informed of their eligibility for the nested qualitative component by the MDR-TBKids clinical staff. Those who expressed interest in participating were then approached by the graduate socio-behavioural science research assistants (KM and SJ) for consenting and data collection.

### Data collection

We conducted in-depth qualitative interviews with participants and/or their caregivers (depending on the child’s age and willingness to express their experiences independently) between 29 April 2021 and 23 September 2021. The interviews were serial, usually involving three interactions over the course of multiple weeks so that we could understand the children’s experiences over the course of their treatment duration. Each interaction was approximately 45–60 minutes in duration. These were audio-recorded, and the data collectors took detailed field notes, which were used to debrief with senior socio-behavioural scientists (AM, DTW & GH) on a weekly basis and thereby inform the case descriptions and subsequent interactions’ follow-up questions. Data collectors were post-graduate socio-behavioral scientists who were trained on the protocol and had experience in conducting research focused on children with TB in similar contexts. The discussion guide included topic areas on (a) household and socio-economic backgrounds, (b) MDR-TB narratives of illness and treatment journeys and, (c) children’s psychosocial experiences of MDR-TB. Interviews were conducted in participants’ preferred language (Xhosa, Afrikaans, or English) in a private space at the site office, Tygerberg.

### Data analysis

After each interaction had been concluded with child-caregiver dyads, we processed data by writing comprehensive case descriptions from audio-recordings of the interviews. These case descriptions were compiled by combining field notes with direct illustrative quotes of participants’ experiences of MDR-TB treatment and care identified through the debriefing process. The detailed case descriptions were refined through multiple revisions and re-engagement with collected data (between authors AM, DTW, JAS, and GH). Case descriptions of all three interactions with each child-caregiver dyad were then combined into a case file. The case files were the ultimate source for our deductive thematic analysis of children and their caregivers’ experiences of MDR-TB.

### Ethics

The study received ethics approval from the Stellenbosch University Health Research Ethics Committee (HREC; N20/09/102). Written informed consent was obtained from the parent/guardian of each child participant. Caregivers provided written informed consent before the start of data collection, with children >7 years old providing additional informed assent. Participants’ identifiable information was replaced and assigned with pseudonyms/participant IDs (PIDs). Only authors have access to information that could identify individual participants during and after data collection – strictly following HREC guidelines to maintain confidentiality. We stored hard copies of data in locked fireproof cupboards at our institution’s offices. Electronic data was encrypted and stored on password-protected computers, backed up in a secured server provided by our institution.

## Findings

We included 17 children in the analysis with ages at enrolment ranging from 1 to 14 years: 11 males and 6 females – Table 1. Eight children had been initially hospitalized for 1–8 months, while 9 received ambulatory care since diagnosis. By the time of data collection completion, some children were completing their MDR-TB treatment. Some children experienced interruptions from treatment in between, which led to incomplete or prolonged treatment duration during our study.

**Table 1:** Characteristics of children with MDR-TB who completed treatment and participated in an in-depth survey study during the COVID-19 period.

Demographics			Treatment timelines				
Age	Sex	Language	Date of diagnosis (M/Y)	Treatment initiation (D/M/Y)	Treatment completion (D/M/Y)	Duration of hospitalisation	Duration of treatment
11	M	Afr	Oct 2018	31 Oct 2018	15 Oct 2019	–	12 months
4	M	Eng	Jan 2020	02 Jan 2020	02 Nov 2020	–	10 months
5	M	Afr	Jan 2018	15 Jan 2018	15 Apr 2019	8 months	15 months
3	F	Afr	Jan 2021	02 Feb 2021	01 Nov 2021	–	9 months
5	M	Afr	Jan 2019	20 Jan 2019	26 Nov 2019	–	11 months
14	F	Xho	May 2019	31 May 2019	31 May 2020	7 months	12 months
3	M	Eng	Nov 2018	15 Nov 2018	15 Feb 2020	1 month	16 months
5	M	Xho	Aug 2018	01 Sep 2018	20 May 2019	–	9 months
4	F	Afr	Aug 2019	12 Sep 2019	12 Dec 2020	–	15 months
5	M	Xho	Sep 2019	03 Sep 2019	–	–	–
2	F	Afr	Feb 2021	16 Feb 2021	15 Nov 2021	7 months	9 months
4	M	Xho	Aug 2019	10 Aug 2019	09 Nov 2020	5 months	15 months
3	F	Xho	Feb 2020	13 Feb 2020	12 May 2021	6 months	15 months
4	M	Xho	July 2019	09 Jul 2019	08 Apr 2020	–	9 months
3	M	Xho	Aug 2019	09 Aug 2019	08 Apr 2020	–	8 months
4	M	Xho	Mar 2018	06 Mar 2018	05 May 2019	8 months	14 months
3	F	Xho	Apr 2019	06 Apr 2019	05 Jan 2020	5 months	9 months

(i) Afr = Afrikaans; Eng = English; Xho = Xhosa (ii) – = not applicable.

### Pathways to care and routes to healthcare services

#### Delayed diagnosis.

We found that more than half of the child participants experienced delays in being diagnosed with MDR-TB. Caregivers reported that their children were repeatedly referred between healthcare facilities prior to their diagnosis. For example, a caregiver whose child was infected by her sister (caregiver’s sister) who was lost to follow-up from TB treatment multiple times reported that:

“We took him to the clinic [for swollen glands]. The nurses sent us to Tygerberg Hospital. In Tygerberg, they gave me a letter telling me to take him to the day hospital. When I got there, they gave me another letter, referring him back to Tygerberg. There, they told me he has [MDR-] TB” (Caregiver of a 4-year-old male).

Caregivers attributed delayed diagnosis to poor healthcare services they experienced at the clinic. One caregiver whose family has a history of people with TB reported that:

“When I was diagnosed with MDR-TB, I asked my mother to get my child tested but they gave her difficulties at the clinic. They refused to test the child for MDR-TB. It is after I went to Worcester hospital [then] they called and ask[ed] them to test the child. That’s when he was referred to Tygerberg Hospital and that’s when he was diagnosed with MDR-TB” (Caregiver of a 2-year-old female).

#### Seeking supplementary care.

Some caregivers reported that after delays in diagnosis, they opted to seek supplementary help from spiritual healers or traditional doctors. One caregiver told us that:

“After multiple visits to the clinic, my child wasn’t getting better, so I took the child to the spiritual healer to be fortified from bad spirits. We received spiritual beads from the healer” (Caregiver of a 4-year-old male).

Other caregivers reported that, even after their child’s diagnosis and treatment initiation, they sought “user-friendly” medication, that might ease the administration process. One caregiver said that:

“I went to a very good pharmacist and told them [my child] had MDR-TB and I’m struggling with the medication, the pills are difficult to crush. He said there is syrup but it’s very expensive. I can’t afford it” (Caregiver of a 5-year-old male).

### Diagnostic experiences

Children and their caregivers experienced emotional distress upon learning about the child’s MDR-TB diagnosis. Some caregivers reported that their children had to be hospitalized immediately after diagnosis which resulted in child-caregiver separation. For example, one caregiver reported that:

“I was in the isolation room next to the reception. I could hear them [clinic staff] talking about my child having MDR-TB. So, the doctor came in later to explain that I had to leave because they were transferring my child to another hospital. That was the worst feeling ever” (Caregiver of a 4-year-old male).

For some caregivers, prior experience of loss exacerbated their emotional distress at learning that their child had MDR-TB.

“It’s these kinds of things [child’s diagnosis] that kept me up at night. I couldn’t sleep because I was constantly thinking about it. I once had a child that died, and nobody cared. I’d wake up with a fright and sit up all night watching over [my child] to make sure that he’s alive” (Caregiver of a 5-year-old male).

### Treatment experiences

Caregivers told us that their children’s treatment journeys were unpleasant. Children and their caregivers had challenges administering the medicines and managing side effects interfacing with adherence over the course of the treatment.

#### Administration.

Caregivers reported that they experienced challenges with administering MDR-TB medication to their children because they often resisted medication ingestion as they struggled to swallow pills. At times, children would spit out the medication due to its bad taste. For example, a caregiver of a 3-year-old child reported that:

“He [the child] never liked pills at all. He would spit them. He’d even cry while I was still preparing to give him his medication. This happened to a point whereby if you were able to absorb the child’s illness to yourself, I would do so.”

Caregivers said that they would prefer treatment to be made available in child-friendly formulations to mitigate administration difficulties. For example, a caregiver to 3- and 4-year-old children said that:

“I really tried to administer medication as consistently as I could, but it wasn’t easy at all. If only the pills in those bottles were combined into one syrup form of medication, I believe things would have been easier because its young children that had to take them.”

Caregivers reported that when their children refused to take medication, they would mix it with dairy products to dissolve pills and mask the taste. One of the caregivers told us that:

“I would use yogurt and put the granules in the yogurt, and now that I have the yogurt, and he opens his mouth to eat the yogurt, I would give him [mix] the medicine. But with the honey, it went much better” (Caregiver of a 5-year-old male).

Caregivers reported that when there were limited products to mix with pills, they would hit their children – out of frustration – to force medication ingestion. This often led to feelings of guilt and shame among caregivers. For example, a caregiver who had to administer MDR-TB medication to two children reported that:

“At times, they [the children] would miss it [medication] because they gave me hard times because they didn’t like it. It wasn’t easy, at times I had to hit them, but I would feel pity for them, and that made me feel so awful” (Caregiver of a 4- and 3-year-old males).

Another caregiver who had a similar experience told us that using force to give medication to her child was the only option. She told us that:

“I struggled to get her to open her mouth to give the medication. That’s why sometimes, when I pushed her mouth open, I’d hurt [injure] her mouth because of the force I used to keep her mouth open, to give her medication. I used to hold her nose closed [to her breathing in the hope she would open her mouth, providing an opportunity to administer the medication] but it didn’t work, she would pass out” (Caregiver of a 4-year-old female).

#### Side effects.

Children and their caregivers reported that children suffered from symptoms such as skin rash, sore feet, and nausea/vomiting. They attributed these symptoms to the side effects of MDR-TB medication. A child who experienced delayed diagnosis and expressed her feelings of distress upon hearing about her MDR-TB diagnosis told us that:

“I became very nauseous when I took those pills. They taste very bad and make me feel very sick” (14-year-old female).

In the same discussion with the child, her caregiver added that:

“At Brooklyn Chest [hospital], she [my child] used to have a painful and leaking ear. That happened when she started with treatment” (Caregiver of a 14-year-old female).

However, the painful and leaking that arose during the treatment phase cannot be attributed as an adverse effect of MDR-TB medication as insinuated by the caregiver.

Another caregiver whose child was hospitalised for three months and later discharged to complete treatment at home described the side effects of medication experienced by her child.

“There were other small pills that were required for him to take at night because they cause [stomach] cramps. So, I always had to buy yogurt to mix with them [granules] so that the taste or pain won’t be that much. This was throughout the hospitalisation period and when he came out of the hospital” (Caregiver of a 4-year-old male).

One caregiver explained how the side effects of medication negatively impacted her psychological well-being.

“The side effects were very extreme on Khaya because I remember at some point, they caused him swollen eyes and confusion. I was distraught and so was he though he couldn’t say it” (Caregiver of a 3-year-old male).

### Impact of diagnosis and treatment on families

#### Strain on household resources.

The illness journeys of children with MDR-TB had adverse impacts on their households’ management of resources. Families had to adjust how they managed income shortly after the diagnosis and during the treatment journeys of their children. Caregivers reported that they had to allocate funds for transport costs to attend their children’s MDR-TB care appointments and buy nutrients and snacks. One of the caregivers said that:

“[My child] used to be selective when it comes to what she eats. Sometimes we would not be able to meet her needs. I’d have to go borrow money I can’t even afford to pay back because she needed to have fruits, yogurt, and milk to mix with medication. We couldn’t eat as we used to. What became worse is that my boyfriend stopped working during that time” (Caregiver of a 4-year-old female).

Another caregiver reported that she had to sacrifice the little she had to attend to the child’s needs during his early days of hospitalisation.

“I had to work so carefully with my pension money so that I have money just to buy nice things and travel to the hospital, otherwise I would have to borrow money from the moneylenders, and I don’t want that” (Caregiver of an 11-year-old male).

#### Competing responsibilities.

Caregivers reported that they had to divert attention and resources from other responsibilities such as work and/or caring for other children in the household and give it to their children with MDR-TB. Some caregivers reported that they had to give up their jobs to provide proper care to their children. For example, one caregiver told us that:

“I had to drop off at work and look after her because [the child’s nannies] got tired. You’d ask someone to look after her and you’d be told the child isn’t well. So, at work, I had to submit daily or weekly excuses reporting that the child is not well [that] led to the loss of income, so I decided to permanently resign and look after her” (Caregiver of a 3-year-old female).

One of the caregivers whose child had been hospitalised shortly after diagnosis and discharged a few weeks later reported that:

“When the child was hospitalised, I had to go back and forth to the hospital which was very difficult because there were other children I [had to] take care of at home. I had to leave them behind and attend to [my child with MDR-TB]. I had to send [my other children] to my aunt for that period” (Caregiver of a 4-year-old male).

#### Stigma.

Children and their families also faced stigma from their neighbours for having MDR-TB. One caregiver, whose father had MDR-TB, reported that community health workers who did regular home visits for her father unintentionally disclosed to others that someone had MDR-TB in their household. She reported that everyone knew about her child’s and father’s MDR-TB illnesses. She told us that:

“What I’ve noticed is that my children were mistreated in the community because you can’t be completely secretive that they have TB. Community members didn’t want our children to play with theirs [because] you know TB can be spread through coughing” (Caregiver to 3- and 4-year-old males).

In one case, a caregiver reported that she felt discriminated at the clinic because her child had MDR-TB.

“Nurses and other health workers didn’t want to go into the TB room where our children were [during check-ups]. It was very painful that we had to give doses to our children by ourselves because nurses said they didn’t want to be infected [by TB]” (Caregiver of a 4-year-old male).

Another caregiver reported that her family is known to have a history of people with TB in the household. She reported that when her child had MDR-TB, they were discriminated against by neighbours. She told us about one incident:

“My neighbour once came to my house after I bought a cool drink. They asked that I pour them a drink. I gave them my glass which I had not used at the time, but they refused it. They said the reason is because I have TB. I was so hurt but now I ignore them” (Caregiver of a 3-year-old female).

### Long-term impact beyond end of treatment

#### Physical impact.

Other caregivers reported that the diagnosis and treatment of their children with MDR-TB had a long-term impact on the child’s physical well-being (e.g., temporary paralysis, slow child development, speech capacity, and shortness of breath). This was more commonly reported by caregivers of children who were diagnosed with MDR-TB at a young age (i.e., less than 3 years). For example, one caregiver said that:

“He doesn’t see properly. If you take a closer look to his eyes or say you take him outside, he can’t stand seeing brightness. Even when he’s staring at something, he closes one eye, he can’t open both at the same time. I thought it was temporary since he recently came out of hospital, but it doesn’t seem to stop” (Caregiver to a 4-year-old male).

Another caregiver of two children affected by MDR-TB described the impact the illness had on the development of their children.

“[MDR] TB really affected his speech capacity. He is older than [his younger brother] but can’t articulate words properly. He stutters. He has that thing of a slow learner or grasping things slow. He completed his treatment, but I was referred to a day hospital for physiotherapy because his other body side [pointing a left arm and leg on a body map activity] was paralyzed” (Caregiver of 3- and 4-year-old males).

## Discussion

We described the experiences of children affected by MDR-TB in a high TB-burden setting. We reported children’s pathways to care and routes to healthcare services prior to their diagnosis, during illness and treatment journeys, and beyond the end of treatment. We found that children and their caregivers encountered multifactorial challenges such as delayed diagnosis due to back-and-forth referrals between facilities, emotional distress upon learning about the child’s MDR-TB diagnosis, and children resisting medication because of poor taste and acceptability. Caregivers had to physically force children to take medication which led to feelings of guilt. Families gave up jobs and ignored other household responsibilities to care for the needs of children with MDR-TB. Families further experienced stigma and MDR-TB had long-term impact on the physical well-being of some children. Our data suggest that children need better medical support.

Participants in our study attributed delays in diagnosis to back-and-forth referrals between healthcare facilities due to healthcare negligence, although in reality it could be due to time to results of special investigations, such as mycobacterial culture and drug susceptibility test results [29]. A qualitative study exploring DR-TB treatment challenges among children found that lack of public health resources, insufficient diagnostic tools, and inability to expectorate sputum remained the leading causes of delayed MDR-TB diagnosis in children [8]. Other factors contributing to delayed diagnosis included health providers’ failure to test for TB/MDR-TB at initial health contact, or incorrectly ascribing symptoms to HIV or other medical conditions [30,31].

We found that delayed diagnosis led to caregivers and their children seeking alternative care such as spiritual healing or traditional medicine. In the eastern parts of Africa, patients initially sought care from traditional healers due to their cultural beliefs [32,33]. The perceived association between MDR-TB and witchcraft has also been reported in studies carried out in Ghana and Kenya [34,35]. In India, patients who were uncertain of the cause of their illness delayed health-seeking initiatives or visited multiple providers prior to going to the public health facility where MDR-TB services could be accessed [36].

In studies conducted in sub-Saharan Africa, participants spoke about the range of emotions they experienced on receiving an MDR-TB diagnosis [8,23,37]. The feeling of distress and hopelessness were prominently noted in these cases [8,23]. MDR-TB diagnosis, despite being devastating for caregivers and their children, also brought a sense of relief because it provided clarity and understanding of their condition [31,32]. Knowing what was wrong helped them move forward with a better sense of direction [31,32]. In our study, children and their caregivers suffered unpleasant emotional reaction upon learning about the child’s MDR-TB diagnosis and that was further exacerbated by immediate hospitalization in some cases.

MDR-TB treatment (which in some patients involved long-term hospitalisation) caused treatment fatigue and burnout among caregivers and/or children during their illness journeys [22,38]. Similar to our findings, children and their caregivers could not endure the burden of long-term MDR-TB treatment – the struggle to administer and ingest medication led to emotional distress and occasional missed doses. Complex challenges around administering MDR-TB medication led caregivers to bribe, threaten, and/or trick children into ingesting medication [9,25]. In our study, caregivers had to mix medication (to mask taste) with dairy products or “physically force” children to ingest pills which caused the overwhelming feeling of guilt to caregivers. Children and caregivers complained about bitter-tasting medication and the adverse effects of MDR-TB treatment [8,21]. The adverse events included, short-term impaired vision, pain in legs, skin colour darkening, nausea and vomiting [8,21]. We found similar trends in our study; older children reported that they felt nauseous and bad taste after taking medication which led to resisting pill intake.

MDR-TB further constitutes a substantial financial burden on both affected families and the health system [14]. Catastrophic costs related to care and emotional distress potentially compromise caregivers’ abilities to provide adequate care for their children with MDR-TB [39]. At the household level, health-related costs such as regular visits to hospitals and buying dairy products to mix with medication are a substantial financial burden [14,40]. In our study, these challenges were further complicated; caregivers had to juggle between prioritising their children’s MDR-TB care and other household priorities such as getting by and/or caring for other children.

In qualitative studies conducted in Africa, caregivers described how stigma, shame, and lack of social support by the community have hindered their ability to ensure their children receive MDR-TB care [23,41]. Stigma in the context of MDR-TB negatively impacts the patients in accessing healthcare facilities in their neighbourhood in fear of social seclusion and/or rejection from family members, friends, and neighbours [41]. None of our participants’ adherence to MDR-TB treatment was interrupted by stigma, contrary to findings in other studies [23,41].

MDR-TB has been reported to have long-term impact on children and their families. A systematic review and meta-analysis on MDR-TB prevalence found that respiratory and hearing loss were the most common type of long-term sequelae among patients treated for MDR-TB [42]. We had fewer cases of children who experienced temporary paralysis, slow child development, speech capacity, and shortness of breath beyond end of their MDR-TB care, as reported by their caregivers. There are increasing attention on the call for improved diagnostics, shorter treatment duration, and injectable-free, and palatable paediatric formulations for children with MDR-TB [15–18,24]. However, as demonstrated in our study, if the psychosocial context to administration remains unchanged, these new developments are unlikely to have optimal gains.

The strengths of our study include that we drew on longitudinal, in-depth data allowing for rich, nuanced descriptions and a diverse sample of children of different ages in a high TB, resource-constrained setting. Extrapolation from these qualitative data to other settings should be made with caution to account for local differences. The data were also collected prior to the availability of bedaquiline and related recent regimen innovations. However, these are still not widely available in many contexts and therefore these findings remain valuable.

Our data show that there are still gaps in the South African healthcare system, with MDR-TB care lacking psychosocial support services needed before diagnosis, during illness and treatment journeys, and beyond the end of treatment. Caregivers play an essential role in the success and adherence of MDR-TB care for children. Family counselling in households affected by MDR-TB is essential to counter emotional distress experienced by caregivers and their children with MDR-TB. Multi-level interventions should focus on child-caregiver-centred approaches that provide psychosocial support during children’s MDR-TB illness and treatment journeys. Future studies should prioritise the collection of similar data from multiple settings, disaggregating by child age, developmental, and schooling/social stage, and alongside the implementation of novel regimens to ensure that children’s MDR-TB-associated morbidity is adequately included in policy and programme decision-making.
